# Extremely low *Helicobacter pylori* prevalence in North Sulawesi, Indonesia and identification of a Maori-tribe type strain: a cross sectional study

**DOI:** 10.1186/s13099-014-0042-0

**Published:** 2014-09-30

**Authors:** Muhammad Miftahussurur, Josef Tuda, Rumiko Suzuki, Yasutoshi Kido, Fumihiko Kawamoto, Miyuki Matsuda, Indah S Tantular, Suhintam Pusarawati, Nasronudin, Paul N Harijanto, Yoshio Yamaoka

**Affiliations:** Department of Environmental and Preventive Medicine, Oita University Faculty of Medicine, 1-1 Idaigaoka, Hasama-machi, Yufu-City, Oita 879-5593 Japan; Gastroentero-Hepatology Division, Department of Internal Medicine, Airlangga University Faculty of Medicine, Surabaya, 60131 Indonesia; Department of Parasitology, Faculty of Medicine, Sam Ratulangi University, Manado, 95115 Indonesia; Institute of Tropical Disease, Airlangga University, Surabaya, 60115 Indonesia; Department of Internal Medicine, Public Hospital of Bethesda Tomohon, Minahasa, 93562 Indonesia; Department of Medicine-Gastroenterology, Baylor College of Medicine and Michael E. Debakey Veterans Affairs Medical Center, 2002 Holcombe Blvd, Houston, Texas 77030 USA

**Keywords:** *Helicobacter pylori*, Maori, Indonesia

## Abstract

**Background:**

Sulawesi in Indonesia has a unique geographical profile with assumed separation from Sundaland. Studies of *Helicobacter pylori* in this region are rare due to the region’s rural location and lack of endoscopy equipment. Indirect methods are, therefore, the most appropriate for measuring *H. pylori* infection in these areas; with the disposable gastric brush test, we can obtain gastric juice as well as small gastric tissue samples for *H. pylori* culture. We investigated the prevalence of *H. pylori* infection and evaluated human migration patterns in the remote areas of North Sulawesi.

**Methods:**

We recruited a total of 251 consecutive adult volunteers and 131 elementary school children. *H. pylori* infection was determined by urine antibody test. A gastric brush test was used to culture *H. pylori*. We used next-generation and polymerase chain reaction based sequencing to determine virulence factors and multi-locus sequence typing (MLST).

**Results:**

The overall *H. pylori* prevalence was only 14.3% for adults and 3.8% for children, and 13.6% and 16.7% in Minahasanese and Mongondownese participants, respectively. We isolated a single *H. pylori* strain, termed -Manado-1. Manado-1 was East Asian type *cagA* (ABD type), *vacA* s1c-m1b, *iceA1* positive/*iceA2* negative, *jhp0562*-positive/*β-(1,3) galT*-negative, *oipA* “on”, and *dupA*-negative. Phylogenetic analyses showed the strain to be hspMaori type, a major type observed in native Taiwanese and Maori tribes.

**Conclusions:**

Our data support that very low *H. pylori* infection prevalence in Indonesia. Identification of hspMaori type *H. pylori* in North Sulawesi may support the hypothesis that North Sulawesi people migrated from north.

## Background

Southeast Asia is a culturally-diverse region enriched by multiple ethnicities and indigenous communities [1]. Indonesia, a developing country at the southeastern tip of mainland Asia and Oceania, is an archipelago of more than 13,600 islands; it has more than 1,000 ethnic and sub-ethnic groups delineated by the Wallace Line, a faunal boundary that separates Asian and Australian ecozones and organisms. Indonesia has more than 730 indigenous languages, most of them belonging to the geographically dispersed Austronesian language family [2]. The age-standardized incidence rate of gastric cancer in Indonesia is reportedly 2.8/100,000, relatively low among Asian countries (International Agency for Research on Cancer; GLOBOCAN2012, http://globocan.iarc.fr/).

More than half of the world’s population is infected with *Helicobacter pylori*, a Gram-negative bacterium etiologically associated with peptic ulcer disease, gastric adenocarcinoma, and primary gastric B-cell lymphoma [3]. Unlike several Southeast Asian countries with high *H. pylori* infection prevalence such as Thailand and the Philippines (54.1 to 76.1% and 60%, respectively) [4,5], several studies have reported low prevalence in Indonesia [6-13]. However these studies primarily investigated Javanese populations, the major ethnic group in Indonesia.

North Sulawesi is an Indonesian province in the northernmost part of Sulawesi Island with Manado as its capital city of; it is composed of 15 districts, with Minahasanese being the predominant tribe. There are currently no detailed data about *H. pylori* infection prevalence in minor ethnic groups in North Sulawesi. This is partly due to a lack of endoscopy systems in remote areas of North Sulawesi. Although invasive, endoscopy gives more information and allows culture of *H. pylori*. We believe that indirect (non-invasive) methods such as the rapid urine test are the best choice for measuring *H. pylori* infection in these areas. Another test option is the disposable gastric brush, which can obtain gastric juice and small gastric tissues for *H. pylori* culture*.* This study therefore investigated the prevalence of *H. pylori* infection in the remote areas of North Sulawesi using rapid urine and gastric brush tests.

*H. pylori* strains from different geographical areas exhibit clear phylogeographic features and *H. pylori* population genetics studies can provide information about the migration of human populations [14-17]. Importantly, North Sulawesi has a unique geographical profile: although the central and western sections of the Indonesian archipelago were connected by dry land to the Asian mainland (Sundaland), Papua (New Guinea) was linked with Australia into a single continent (Sahul) about 60,000 years ago (60 ka). Sulawesi and the Philippines (except for Palawan) are assumed to be zoogeographically separated from Sundaland, a hypothesis supported by distribution patterns of mammals and birds [18]. Several authors believe that the ancestors of the North Sulawesi peoples came from the north because the traditions, manners, customs, and languages of the Minahasanese and Mongondownese people belong to the Philippine language group [19].

We previously showed that *H. pylori* could be divided into seven major populations (hpAfrica1, hpAfrica2, hpNEAfrica, hpEurope, hpAsia2, hpEastAsia, and hpSahul) based on multi locus sequence typing (MLST) using sequences of seven housekeeping genes (*atpA*, *efp*, *mutY*, *ppa*, *trpC*, *ureI*, and *yphC*) [14,15,20]. hpEastAsia is divided into three subpopulations: hspMaori in native Taiwanese, Polynesian, and Melanesian people. Our recent analyses showed that hspMaori populations migrated from Taiwan through the Pacific around 5,000 years ago [20]. However, the study did not include *H. pylori* strains from Sulawesi or several islands in eastern Indonesia. It is important to clarify this migration model to support our hypothesis that Indonesia could be a junction of human migration and that waves of human migration occurred more than twice in this region. Therefore, a second purpose of this study was to use *H. pylori* as a tool to evaluate human migration patterns.

## Results

### Study population and *H. pylori* infection rate in North Sulawesi

**Figure 1:**
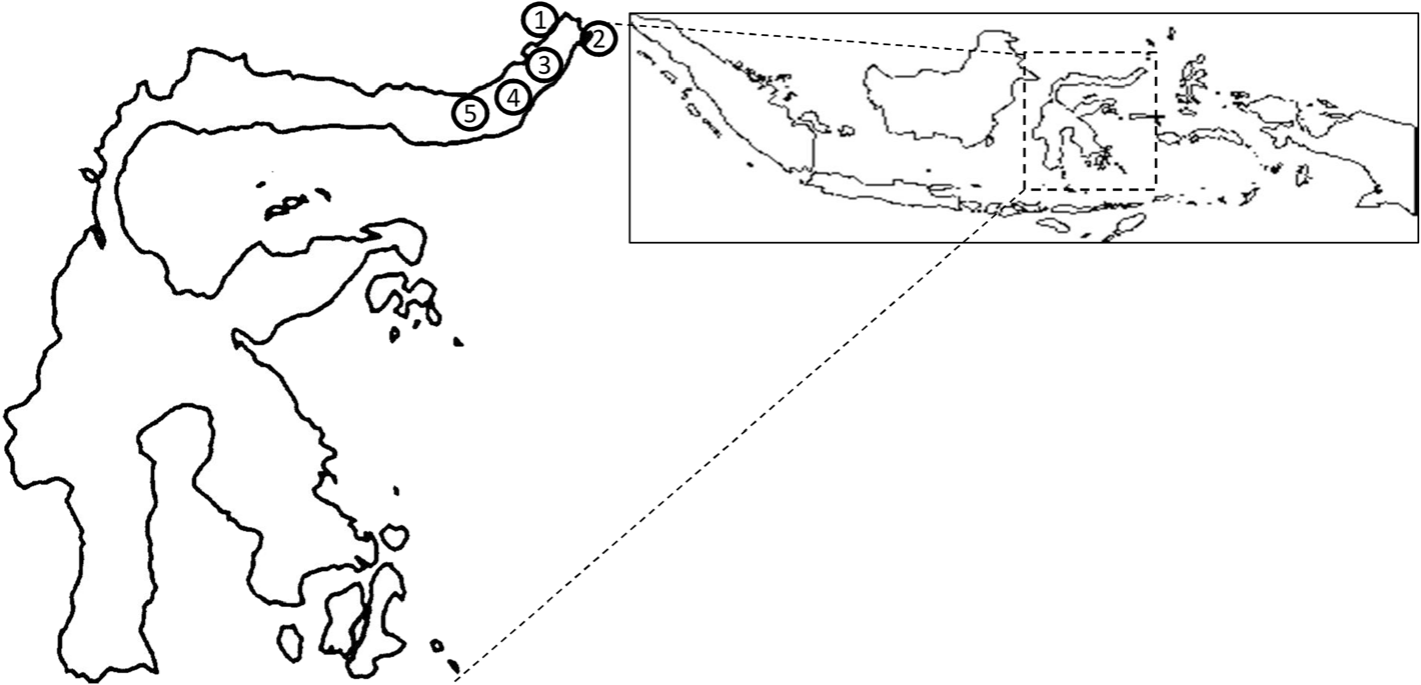
**Collecting areas in the North Sulawesi province.** Urine was collected from a total of 251 consecutive volunteers from the (1) Wori, (2) Bitung, (3) Tomohon, (4) Amurang, and (5) Kotamobagu regions in the North Sulawesi province.

We performed three surveys in North Sulawesi province from April 30 to May 1, 2011, January 30 to February 3, 2012, and July 23-24, 2012. Consecutive adult volunteers were enrolled in each survey. In total, 251 volunteers (146 women and 105 men; mean age, 46.2 ± 19.5 years old; range, 14–88 years) were included and provided urine samples. The study consisted of 147 Minahasanese subjects, 90 Mongondownese, six Javanese, three Gorontalonese, one Makassarese, one Ternatenese, one Sangirese, one Balinese, and one Bataknese subject. A map of the collection area is shown in Figure 1. We also obtained urine samples from 131 elementary school children (71 girls and boys; mean age 8.47 ± 1.64 years; range, 6–12 years) in Wori during the July 23-24, 2012 survey.

**Figure 2:**
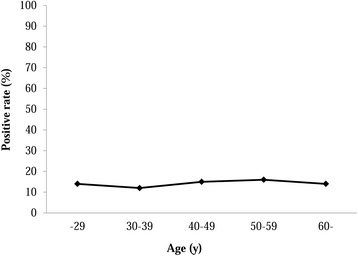
**Prevalence of** ***Helicobacter pylori*** **infection in North Sulawesi by age group.** Subjects with positive rapid urine test (RAPIRUN® *H. pylori* antibody, Otsuka Pharmaceutical Co., Tokyo, Japan) results were considered positive for *H. pylori*.

The population of 251 adult volunteers consisted of 57 subjects aged ≤29 years, 34 subjects aged 30–39 years, 60 subjects aged 40–49 years, 37 subjects aged 50–59 years, and 63 aged ≥60 years old. The *H. pylori* prevalence by age group was 14.0% (8/57), 11.7% (4/34), 15.0% (9/60), 16.2% (6/37), and 14.3% (9/63) respectively, with an overall *H. pylori* based on urine testing of 14.3% (36/251) (Figure 2). There was no statistically significant relationship between *H. pylori* prevalence and age or sex (P = 0.84 and P = 0.69). Only five elementary school children (3.8%) were positive for *H. pylori*, a rate significantly lower than the adult population (P = 0.02).

To confirm the urine test accuracy, 50 subjects were randomly selected among the 251 adult volunteers for *H. pylori* seropositivity serum testing. Three (6.0%) patient serum samples were positive for *H. pylori* antibodies, identical to the urine test findings (100% sensitivity, specificity, and accuracy based on serum *H. pylori* antibody test as a gold standard).

### *H. pylori* infection rates according to ethnicity

Among 147 Minahasanese subjects, 20 (13.6%) were positive for *H. pylori.* Fifteen of 90 (16.7%) Mongondownese were positive for *H. pylori* infection (P = 0.59 compared with Minahasanese). One of six (16.6%) Javanese study subjects was positive for *H. pylori*. In contrast, none of the Gorontalo, Makassarese, Ternatenese, Sangirese, Balinese, or Bataknese study subjects tested positive for *H. pylori* infection.

### Identification of a North Sulawesi *H. pylori* genotype

Among 36 patients infected with *H. pylori*, 6 agreed to undergo gastric brush tests. We successfully cultured *H. pylori* from one patient; we termed the strain Manado-1.

Virulence factor analysis using PCR/PCR-based sequencing showed that the strain was East Asian type *cagA* (ABD type), *vacA* s1c-m1b, *iceA1* positive/*iceA2* negative, *jhp0562*-positive/*β-(1,3) galT*-negative, *oipA* “on” and *dupA*-negative. A Genbank database BLAST search of full-length Manado-1 *cagA* sequences showed greatest nucleotide similarity with strains PHL2 (GU173854.1) and PHL10 (GU173856.1) from the Philippines, with homologies of 97.7% and 97.6%, respectively.

### Manado-1 population type

**Figure 3:**
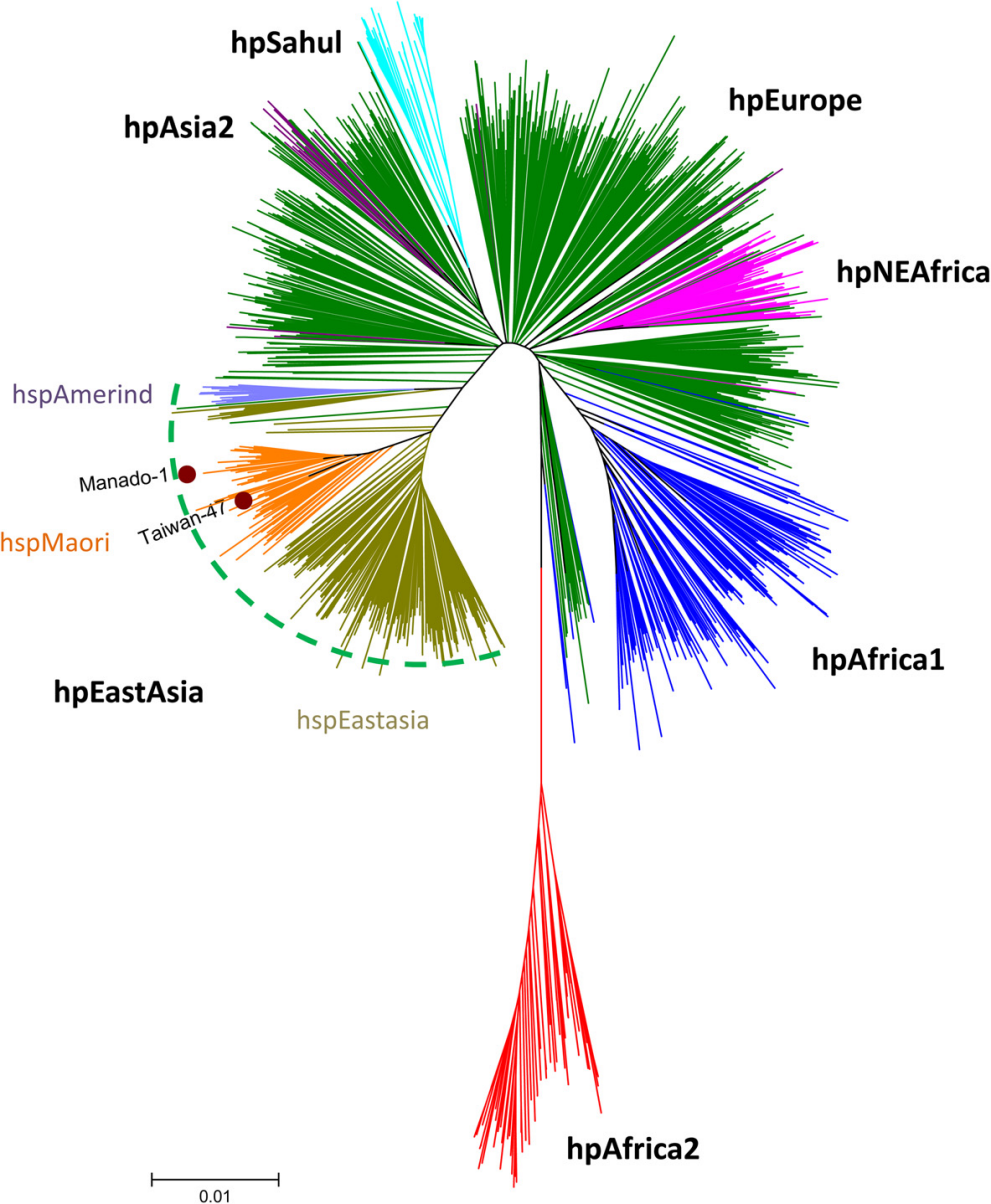
**Phylogenetic tree based on the seven housekeeping genes of** ***H. pylori*** **.** Sequence data sets of the seven housekeeping genes of 1,126 strains with different genotypes were obtained from the pubMLST database (62 from hpAsia2, 493 from hpEurope, 76 from hpNEAfrica, 50 from hpSahul, 28 from hpAfrica2, 279 from hpEastAsia, and 138 from hpAfrica1). The 1,126 reference strains from GenBank, Manado-1, and Taiwan-47 strains are included. Neighbor-joining trees were constructed in MEGA v.5.05 using Kimura-2 parameters. The scale bar indicates genetic distance.

We constructed a phylogenetic tree using MLST sequence data from Manado-1 and 1,231 PubMLST strains. Manado-1 clustered with hspMaori strains (Figure 3). Branch colors represent *H. pylori* populations previously determined using STRUCTURE Bayesian clustering software [16].

We sequenced Manado-1 and a strain isolated from a native Taiwanese participant (Taiwan-47) using the next-generation sequencer. We used next-generation sequencing data to construct 39 and 34 Manado-1 and Taiwan-47 contig sequences, respectively (average sequencing coverage and %GC of 2,455x and 38.9% and 721x and 38.7%, respectively), and extracted protein-coding sequences from the contigs. We then integrated this data with sequence data obtained from GenBank to construct a phylogenetic tree based on the concatenated sequences of 644 orthologous genes shared by all analyzed strains.

**Figure 4:**
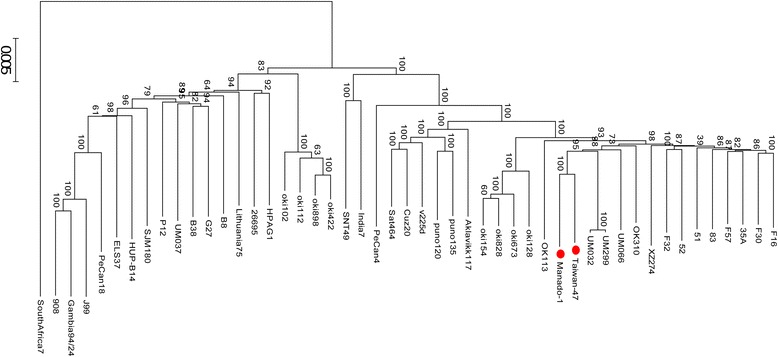
**Phylogenetic tree of Manado-1 and Taiwan-47 with 47 complete genome strains.** This tree was constructed using concatenated sequences of 644 orthologous genes. Manado-1 and Taiwan-47 are marked with red circles. Bootstrap values (percentage) are shown on the branches.

Manado-1 and Taiwan-47 formed a phylogenetic tree sub-branch with Malaysian and some Okinawa strains, which reflects phylogeographic differences from typical East Asian strains (Figure 4).

### Nucleotide sequences

Nucleotide sequence data for strains Manado-1 and Taiwan-47 are available under DDBJ accession numbers AB985740 to AB985753 for MLST, and JRAC00000001-38 and JQNY00000001-58 for the genome data using the next generation sequencer.

## Discussion

We observed extremely low *H. pylori* prevalence in North Sulawesi, Indonesia using RAPIRUN, a rapid immunochromatography method for detection of anti-*H. pylori* IgG in urine; this result is consistent with our previous report of very low prevalence among Javanese [6-12,21]. We recently confirmed the high accuracy of RAPIRUN using five different methods to test *H. pylori* status (culture, rapid urease test, histology, immunohistochemistry, and RAPIRUN) in Surabaya, on Java island in Indonesia [21]. In this study, we also found identical results from urine and serum samples.

Contrary to typical age-related *H. pylori* prevalence patterns in developing countries, where *H. pylori* infections occur earlier in life and with higher frequency [22], we found that only 3.8% of participants with a mean age 8.47 years were infected*.* Even taking into account the possibility of negative rapid urine test results in children based on reports of lower antibody titers than true positive results [23], the prevalence of *H. pylori* in children in North Sulawesi was very low. The transmission routes of *H. pylori* are still not entirely understood, but human-to-human spread through oral-oral or fecal-oral routes are considered the most plausible infection routes [22]. In developing countries, *H. pylori* is mainly transmitted feco-orally, in contrast to gastro-oral transmission in developed countries [24]. The degrees of crowding and contact between family members during are important variables. For example, Malaty et al. reported a 23% *H. pylori* prevalence in children of infected mothers, whereas only a 5% prevalence was observed in children of uninfected mothers [25]. Further studies are necessary to clarify the role of familial clustering in typical age-related prevalence patterns of *H. pylori* infection in North Sulawesi.

Javanese form the predominant ethnic group in Surabaya and have a close genetic relationship and common ancestral history with Melayu populations in Malaysia [26], which also have low *H. pylori* infection rates [1], suggesting that host genetic factors might contribute to reduced *H. pylori* infection susceptibility. However, the Javanese ethnic origins differ from those of Minahasanese, so it is unclear why the *H. pylori* prevalence was very low in both ethnic populations.

Although *H. pylori* was isolated from only one subject in North Sulawesi, it was interesting that the strain was hspMaori, a subpopulation of the East Asian type often isolated from native Taiwanese and Maori tribes as well as some individuals from the Philippines [20]. The Philippines and Sulawesi are neighboring regions assumed to be zoogeographically separated from Sundaland. Therefore, our data support the hypothesis that the Philippines hspMaori type was not only spread directly to the Pacific islands, but also to Sulawesi. Previously, the hpSahul populations were believed to pass through the Pleistocene landmass known as Sundaland (i.e., the Malay peninsula, Sumatra, Java, Borneo, and Bali) 23,000 to 32,000 years ago [20]. Much later, migration of Javanese ancestors through this region likely replaced the indigenous peoples; therefore, hpSahul remained isolated in New Guinea and Australia.

However, differences in *H. pylori* infection rates between this study and data from the Philippines was marked: *H. pylori* prevalence in the Philippines is a reported 60% (31/52) [5]. This difference cannot be explained by sanitary conditions alone, because food hygiene and drinking water quality in the Philippines are better than in Indonesia: approximately 50% of the Indonesian population has basic environmental sanitary conditions, especially in rural areas (UNICEF, http://www.unicef.org/). Two possibilities could explain differences in *H. pylori* infection rates between Minahasanese and Philippinos. First, *H. pylori* prevalence might be low among native Philippinos with hspMaori type. However, it does also not mean that hspMaori *H. pylori* is universally difficult to infect, since *H. pylori* prevalence in native Taiwanese is high [27]. As a former Spanish colony, current Philippino populations may have acquired *H. pylori* through intermarriage of various races and nationalities with indigenous ethnic groups, resulting in reduced hspMaori strains due to competition with novel strains. This theory is supported by the fact that the majority of *CagA* types in the Philippines are Western type [28]. Interestingly, Minahasanese populations also had extensive relations and communications with Western countries, especially the Dutch (350 years ago), but relatively few people are infected with *H. pylori.* Therefore, specific environmental factors including Minahasanese dietary habits, may contribute to reduced *H. pylori* susceptibility in North Sulawesi.

The second possibility to explain different infection rates is that migration waves from Taiwan through the Pacific that distributed hspMaori around 5,000 years ago might not involve Sulawesi island; the original introduction of the Manado-1 strain may have occurred later, which is likely considering that Manado is a coastal city. In this hypothesis, Minahasanese and Philippinos might have different ancestral origins. A larger sample size of *H. pylori* strains isolated from North Sulawesi is necessary to elucidate the origin of *H. pylori* strains in Indonesia.

Only one strain was obtained after culturing, which was one limitation of this study. The lack of frozen conditions during transportation likely contributed to the low culture rate of the gastric brush test. *H. pylori* recovery rates have been reported to be 25% (1 of 4) when stored for 7 days at 4°C, resulting in markedly diminished recovery [29]. In this survey we kept the specimens at 4°C from the time of collection at the rural hospital to the time they were stored at -20°C for a week at Sam Ratulangi University, Manado. The specimens were then sent to Surabaya at room temperature to be kept at -20°C for 3 days at Airlangga University Faculty of Medicine before culture. Future surveys should immediately freeze or culture the fluid that dripped from the brush.

Another limitation of this study was a lack of volunteer medication information. It is possible that we have included patients who had been administered antibiotics, histamine-2 receptor antagonists (H2-blockers), or proton pump inhibitors, which can influence *H. pylori* infection prevalence. However, a previous report found the prevalence of *H. pylori* infection in Indonesia to be quite low (10.2%) even after patients taking proton pump inhibitors were excluded from the study population [10].

## Conclusions

We found extremely low *H. pylori* prevalence in North Sulawesi. We also discovered an hspMaori type strain in North Sulawesi, which was widespread among aboriginal Taiwanese tribes, supporting the hypothesis that North Sulawesi people came from the north, although these findings remain to be confirmed.

## Methods

### *H. pylori* infection status

*H. pylori* status was evaluated with a rapid urine test (RAPIRUN® *H. pylori* antibody, Otsuka Pharmaceutical Co., Tokyo, Japan) according to the manufacturer’s instructions. RAPIRUN has been reported to have high accuracy, with excellent sensitivity, specificity, and accuracy in Japanese (92.0%, 93.1%, and 92.3%, respectively) [30] and Vietnamese populations [31]. Immediately after collection, urine samples were tested for *H. pylori* antibodies. All urine samples were measured and analyzed by a skilled researcher (MM) blinded to subjects’ information.

A disposable extendable oro-gastric brush was constructed on a guide-wire with a handle (modified Baylor Gastric Brush, Tochigi Seiko, Tochigi, Japan). The brush was ~5 mm in diameter and fit within an enlarged distal sheath portion. Withdrawal of the brush into the sheath closed the brush compartment. After administration of topical oral anesthesia, the 5-mm brush assembly was swallowed. Approximately 60 cm from the incisor teeth, the brush was extended and moved back and forth 3–8 cm, three or four times. The brush was then retracted into the protective sleeve and withdrawn from the subject. The brush was placed in a dram vial containing approximately 1 mL of cysteine transport medium with 20% glycerol [32], mixed by shaking, and 100 μL withdrawn for culture. Samples were kept at 4°C and stored at -20°C within a day of collection at Sam Ratulangi University, Manado. The samples were then sent for isolation of *H. pylori* using standard culture methods at the Institute of Tropical Disease, Airlangga University in Surabaya [33]. Culture stocks were sent to Oita University Faculty of Medicine for further analyses.

We also randomly selected 50 subjects for anti-*H. pylori* antibody serum testing: serum samples were transported to the Institute of Tropical Disease at Airlangga University using the same procedure described above. *H. pylori* seropositivity was evaluated using a commercially available ELISA kit (Eiken Co., Ltd., Tokyo, Japan) according to the manufacturer’s instructions.

Informed consent was obtained from all participants, and the protocol was approved by the Ethics Committees of Sam Ratulangi University (Manado, Indonesia) and Oita University Faculty of Medicine (Yufu, Japan).

### *H. pylori* isolation and genotyping

*H. pylori* DNA was extracted using the QIAamp DNA Mini Kit (QIAGEN, Valencia, CA) according to the manufacturer’s directions. Whole-genome sequencing was performed using 90-base paired-end reads on an Illumina HiSeq 2000 next-generation sequencer (Illumina, Inc., San Diego, CA). The nucleotide sequences of *cagA*, *vacA*, *jhp0562, β-(1,3) galT, oipA, iceA,* and *dupA* genes were extracted from the data and confirmed by PCR-based sequencing as described previously [33-39]. Multi locus sequence typing genes; *atpA, efp, mutY, ppa, trpC, ureI*, and *yphC* were also extracted from the next-generation sequencing data and confirmed by PCR-based sequencing as described previously [34].

### Phylogenetic analysis of *H. pylori* strains

A phylogenetic tree was constructed using MLST sequence datasets comprised of seven housekeeping genes from 1,231 strains with different genotypes obtained from the pubMLST database (http://pubmlst.org/). These sequence datasets were integrated with our North Sulawesi sequence data. A neighbor-joining tree was constructed based on the sequence alignment using MEGA v.5.05 (Kimura-2-parameter model) [40,41]. We also sequenced Manado-1 and a strain isolated from a native Taiwanese participant (Taiwan-47) using the next-generation sequencer (HiSeq2000). HiSeq2000 output was integrated into contig sequences by CLC Genomics Workbench 7.0.4. Genomics Workbench was also used for gene prediction and translated to protein sequence using an original perl script. Protein coding genes of 47 *H. pylori* strains whose genomes are publically available were obtained from GenBank. We defined gene groups using OrthoMCL gene clustering software and selected single-copy orthologs from each strain, resulting in a total of 644 gene groups. We aligned gene sequences using MAFFT version 7 (http://mafft.cbrc.jp/alignment/server/), concatenated the alignment using an original perl script, and used MEGA v.5.05 to construct a phylogenetic tree (Poison model, bootstrap 1,000).

### Statistical analysis

Data were analyzed using SPSS, version 19 (SPSS Inc., Chicago, IL, USA). Discrete variables were tested using the chi-square test; continuous variables were tested using Mann-Whitney *U* and *t*-tests. A two-tailed P*-*value <0.05 was considered statistically significant.

## Abbreviations

MLST: Multi-locus sequence typing
PCR: Polymerase chain reaction
